# Capsule-epithelium-fibre unit ultrastructure in the human lens

**DOI:** 10.7150/ijms.82446

**Published:** 2023-02-27

**Authors:** Qingwen Yang, Lingying Ye, Jiasheng Liu, Lei Lin, Wenjing Wang, Mengchao Zhu, Lu Zhang, Jiayi Xiao, Jun Zhang, Jin Li

**Affiliations:** 1 Department of Cataract, Eye Hospital of Wenzhou Medical University, Wenzhou 325000, Zhejiang, China; 2 Laboratory of Retinal Physiology and Disease, Eye Hospital of Wenzhou Medical University, Wenzhou 325027, China.; 3 State Key Laboratory of Ophthalmology, Optometry and Visual Science, Wenzhou 325027, China.

**Keywords:** transmission electron microscopy, ultrastructure, human lens, epithelium-fibre interface, capsule-epithelium-fibre unit

## Abstract

This study aimed to investigate the capsule-epithelium-fibre unit ultrastructure of the human lens, particularly the interfaces of the epithelium with the capsule and the epithelium with the fibre cell. A total of 12 lenses from donor humans who died of trauma without systemic and ocular diseases were investigated by transmission electron microscopy (TEM), combined with immunofluorescence staining for localising certain specific proteins. Some of the results were further studied in the anterior lens capsules of cataract patients. Our results revealed capsule protrusion into the epithelium in some areas and potential processing of capsule components. The young elongating fibre cells directly adjacent to the epithelium with a high stain density strongly expressed CD24. Numerous extracellular vesicles could be seen in the space between human lens epithelial cells (HLECs) and between HLECs and the capsule. Mitophagy and autophagy were also observed in the HLECs. Our research may be beneficial in better understanding the function of the human lens.

## Introduction

The human lens is an important structure, and together with the cornea, it focuses light on the retina to form a real image 1. Millions globally are confronted with blindness and visual impairment due to lens pathologies 2. To address this requires greater investigation of the lens, including its ultrastructure.

The lens is composed of two forms of cells: a monolayer of lens epithelial cells covering the anterior surface, and fibre cells, which constitute the bulk of the tissue. Lens epithelial cells differentiate into lens fibre cells in the bow region throughout the lifetime 3, 4. Significant literature has described the lens ultrastructure 5-9. Normal human lens epithelial cells (HLECs) have complex interdigitating processes along the lateral borders that can extend into the adjacent cell, which appear like intercellular bridges. The basal surface of HLECs towards the lens capsule has numerous small pores and irregular craters with serpentine-like processes 7.

Although the HLEC ultrastructure has been studied extensively, particularly that on the anterior lens capsule (aLC) of cataract patients obtained from routine cataract surgery, little ultrastructure information is available about the capsule-epithelium-fibre unit, particularly the interface between the lens epithelium and capsule and that between the lens epithelium and fibres. Therefore, the current study investigated this structure by transmission electron microscopy (TEM) and immunofluorescence staining.

## Materials and Methods

### Human lenses

In total, 12 donated human lenses of those who had died of trauma without systemic or ocular diseases and ranged in age from 16 months to 67 years were obtained from the Wenzhou Medical University Eye Bank. Additionally, 12 aLCs of cataract patients were obtained from routine cataract surgery through continuous central curvilinear capsulorhexis, which was approximately 5-5.5 mm of the central aLC. The lens information is presented in Table 1.

### Transmission electron microscopy

Vibratome lens sections of approximately 200 μm thickness were cut from fresh lenses using a thin razor blade as the knife. The lens slices were immediately immersion fixed in 0.1 M phosphate buffer (pH 7.4) containing 2% paraformaldehyde (cat. no. 157-8, Electron Microscopy Sciences, Hatfield, PA) and 2% glutaraldehyde (cat. no. 16020, Electron Microscopy Sciences, Hatfield, PA) for 12 h. Following washing with 0.1 M phosphate buffer (pH 7.4), tissues were subsequently postfixed in 1% osmic acid for 1-1.5 h. A series of graded acetone (30%, 50%, 70%, 80%, 90%, and 100%) was used to dehydrate these samples for 15 min in each step and were then transferred to 100% acetone for 15 min. Following rinsing dehydration, the tissues were embedded in Eponate 12 resin for 72 h at 60 °C. The area with regions of greatest interest was pre-screened on one-micron thick sections stained with toluidine blue under a light microscope. Subsequently, 70- to 90-nm sections were collected in 200-mesh grids and counterstained with 5% uranyl acetate and 0.3% lead citrate. The sections were observed under an H-7500 transmission electron microscope at 60 kV. The central aLCs from cataract patients were prepared for TEM with the protocol described above.

### Immunofluorescence

Lens capsule flat mounts or lens sections were fixed with 4% paraformaldehyde for 20 min, washed with phosphate-buffered saline (PBS) three times, and permeabilised with 0.3% Triton X-100 for 40 min. Tissues were blocked in 5% sheep serum and 1% bovine serum albumin dissolved in PBS for 1 h and incubated overnight with primary antibodies at 4 °C. The primary antibodies were used at the following dilutions: SQSTM1/p62 (ab56416, Abcam, Cambridge, UK) at 1:200, CD24 (ab202073, Abcam) at 1:200, CD63 (ab271286, Abcam) at 1:200 and CD81 (ab219209, Abcam) at 1:200. Following three washes in PBS, the tissues were incubated with secondary antibody (Alexa Fluor 488 conjugated anti-rabbit IgG, ab150077, Abcam) at 1:800 for 2 h at room temperature. Cell nuclei were stained with DAPI. Images were obtained using a Zeiss LSM700 confocal microscope.

## Results

### Capsule protruded into the epithelial cells in some regions

**Figure 1:**
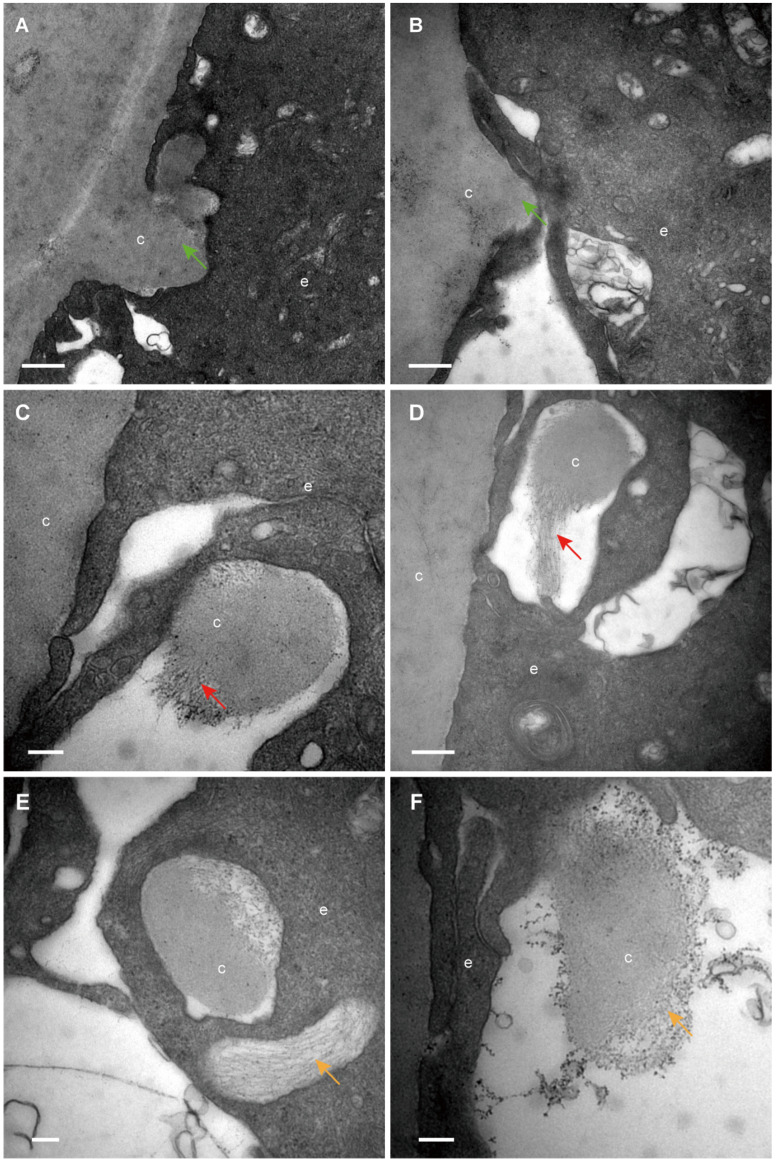
** Different capsule protrusion morphology in a donor lens.** The capsule protrudes into HLECs **(A, B green arrow)**. The protruding capsule is completely enclosed by the epithelial process and is loose on the periphery **(C, D red arrow)**. The filament-like changes of embedded capsule tissue **(E, F, orange arrow)**. Scale bars: A = 1 µm, B = 0.5 µm, C-F = 0.2 µm. All TEM images are from a 51-year-old donor.

**Figure 2:**
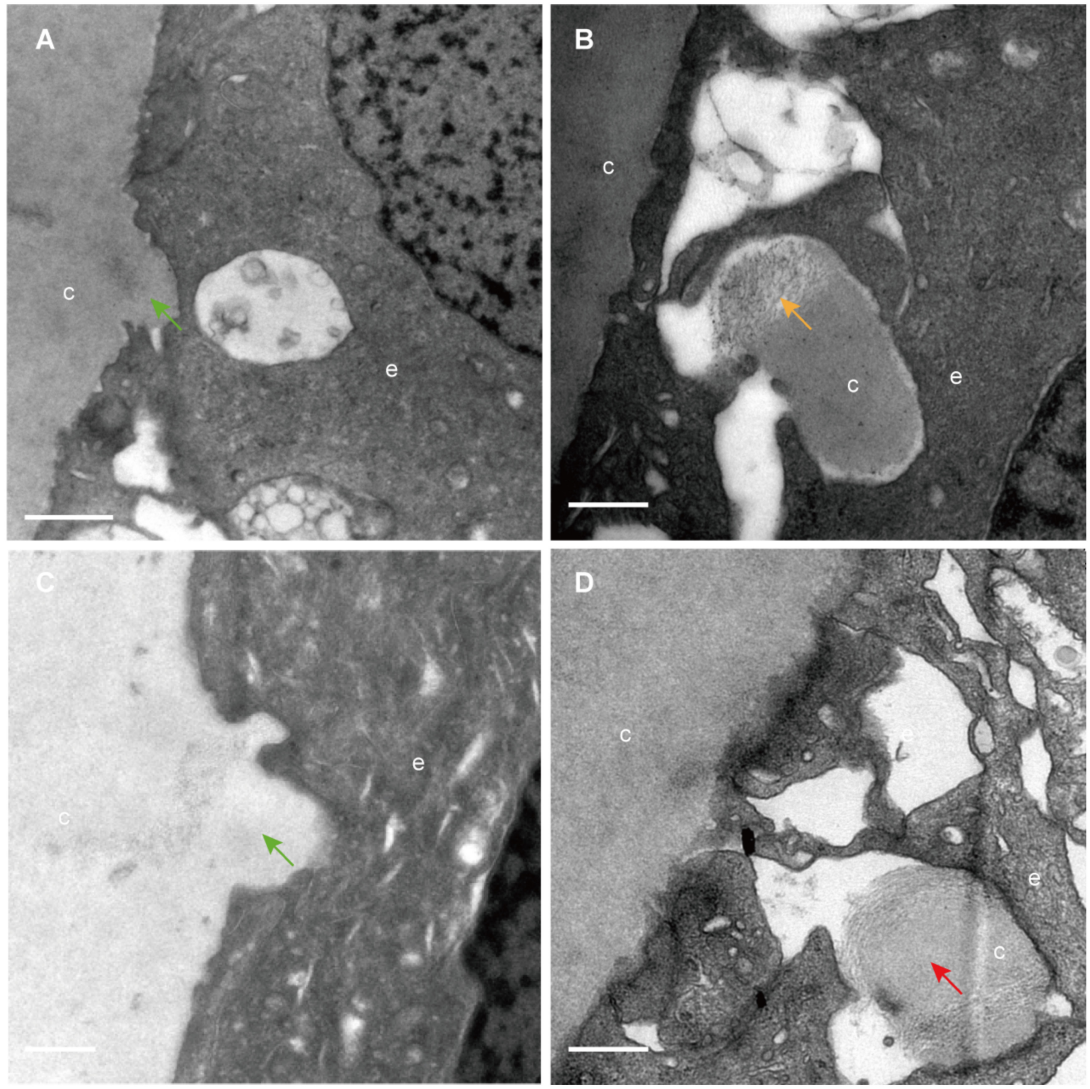
** Capsule protrusions in human lenses of different ages.** Initial capsule protrusions **(A, C green arrow)**, enclosed capsule **(D, red arrow)** and filament-like capsule protrusions **(C, orange arrow)** was observed in different ages of human lenses. Different colours arrow shows different morphology of the protruding capsule. The representative images of the capsule were obtained from a 41-year-old donor **(A)**, a 51-years-old donor **(B)**, a 65-year-old patient with cataract **(C)** and a 67-year-old donor **(D)**. Scale bars: A = 1 µm, B-D = 0.5 µm.

The classical EM study reported that the lens capsule is a uniform and non-cellular structure composed of electron-dense materials, which smoothly covers anterior surfaces of the HLECs 10-13. Interestingly, our results showed that in some cases human lens capsule is not as smooth as reported before. We analysed 12 donated lenses and 12 aLCs of cataract patients obtained from routine cataract surgery ultrastructurally (Table 1), and found that the capsules from 3 (25%) donor lenses and 2 (17%) cataract patients could protrude into the epithelial cells in some unidentified regions but not in the equatorial zone (Figure 1). The protruding capsules appeared to display three different morphological characteristics, with representative TEM images of one donor lens of a 51-year-old shown in Figure 1. Different colours arrow shows different morphology of the protruding capsule. Figure 1A, B illustrates the capsule protruding into the epithelial cells (green arrow). Figure 1C, D shows the protruding capsule completely enclosed by the epithelial processes and had become loose on the periphery (red arrow). Figure 1E, F exhibits the filament-like changes (orange arrow) of the embedded capsule tissue. These three different morphological capsule protrusions were observed in the human lenses of some ages. The detailed information is presented in Table 1. Representative images of the protruding capsule of different ages are shown in Figure 2. The colors of the arrows signify specific features of the protrusions.

### Young elongating fibre cells with high stain density strongly expressed CD24

**Figure 3:**
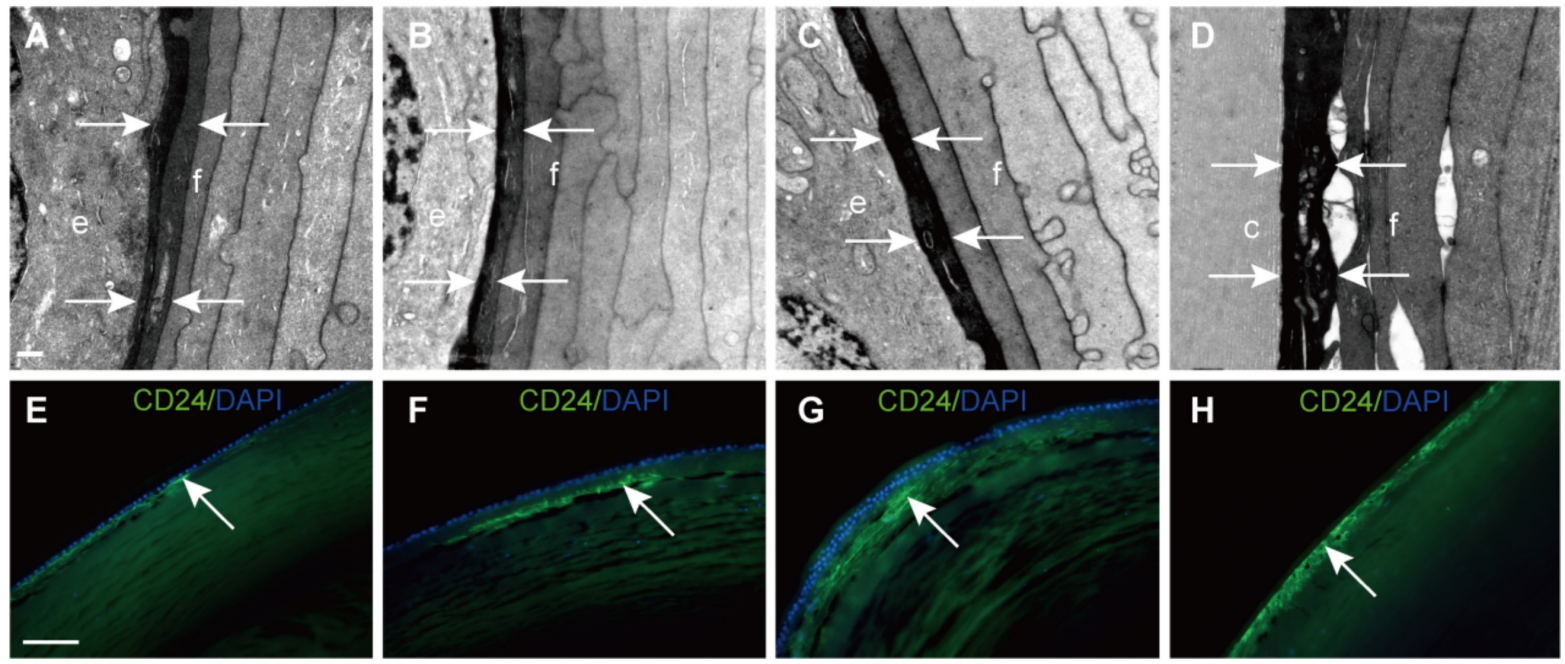
** Electron-dense fibre cells highly expressed CD24 in the epithelium-fibre interface. (A-D)** TEM results showing young elongating fibre cells **(f)** displaying high electron density (white arrows) around the entire lens. **(E-H)** Representative images of immunofluorescent staining of CD24 (green), with CD24 highly expressed in the young elongating fibre cells but absent in the epithelium (white arrow). **(D and H)** are from the posterior capsule. Scale bars: A-D = 0.5 µm. E-H = 20 µm.

In this study, we found that young elongating fibre cells displayed a high electron density in all samples. Representative images are shown in Figure 3A-D. To further explore this structure, CD24 immunofluorescence was performed on vertical sections of the human lens. The results showed that CD24 immunoreactivity was restricted to the narrow area between the epithelial cells and developing fibre cells (Figure 3E-H), in agreement with our TEM results.

### Numerous extracellular vesicles were observed in the space between HLECs and between HLECs and the capsule

**Figure 4:**
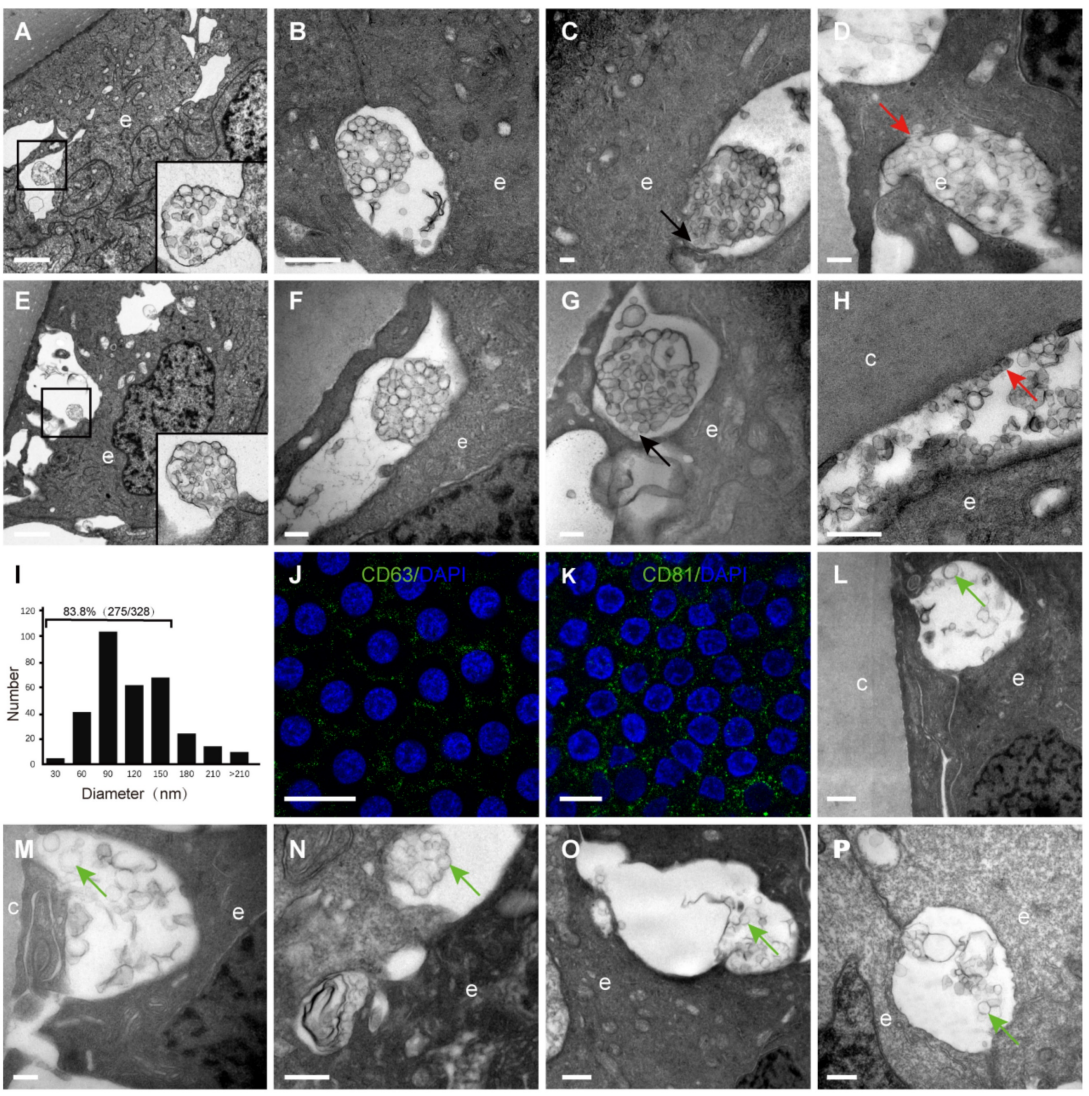
** Extracellular vesicles (EVs) in the human lens.** Extracellular vesicles complex with the membrane continuing to the plasma membrane of the HLECs **(A, E)**. Isolated membrane-bound vesicles with intact membrane **(B, F)**. Isolated membrane-bound vesicles with discontinuous membrane **(C, G)** (black arrow). Smaller vesicles in the extracellular space, some of which are closely attached to the HLECs **(D)** and capsule **(H)** (red arrow). Of the EVs, 83.8% were 30-150 nm in diameter **(I)**. Representative images of immunofluorescent staining of the exosome markers CD81and CD63 (green) in flat-mounted HLECs **(J, K)**. Typical EVs (green arrow) in the aLCs of age-related cataract **(M, L)**, primary acute angle-closure glaucoma complicated cataract **(N)**, uveitis complicated cataract **(O)** and congenital cataract **(P)**. Scale bars: A, E = 2 µm, B = 1 µm, C, D, F, G, M = 0.2 µm, H, L, N-P = 0.5 µm, J, K = 20 µm.

By TEM, different sizes and shapes of extracellular vesicles (EVs) were observed in the extracellular space of the HLECs in all donor samples (Table 1). Four different morphologies of the EVs were observed in the current study, which are summarised in Figure 4: An extracellular membrane-bound vesicular structure was observed at the basal side of the HLECs, the membrane of which appeared to be continuous with the plasma membrane of the HLECs. The majority of these membrane structures were oval shaped, with diameters of 2-3 μm, containing numerous smaller vesicles (Figure 4A); isolated membrane-bound vesicles in the extracellular space with intact membrane (Figure 4B); isolated membrane-bound vesicles in the extracellular space with a discontinuous membrane (Figure 4C); numerous smaller vesicles distributed in the extracellular space, some of which were closely attached to the HLECs (Figure 4D). Similarly, EVs were found in the space between HLECs and the capsule, some of which analysed 328 smaller EVs from 10 TEM images, and their diameters were between 26.8 and 307.3 nm. Interestingly, 83.8% (275/328) of them ranged from 30 to150 nm (Figure 4J). Immunofluorescence results illustrated that exosome markers (CD63 and CD81) were expressed in the HLECs on the lens capsule flat-mount (Figure 4K、L). In addition, these results were further studied in the aLCs in different types of cataract patients after routine cataract surgery, including age-related, complicated and congenital cataracts. Different EV sizes and shapes were also observed in the extracellular space at the basal side of the HLECs. However, EVs in the cataract samples appeared to be more irregular and disorganised, and the four typical morphologies as described above were rarely observed. Representative TEM images are shown in Figure 4L-P.

### Autophagy and mitophagy were observed in HLECs

**Figure 5:**
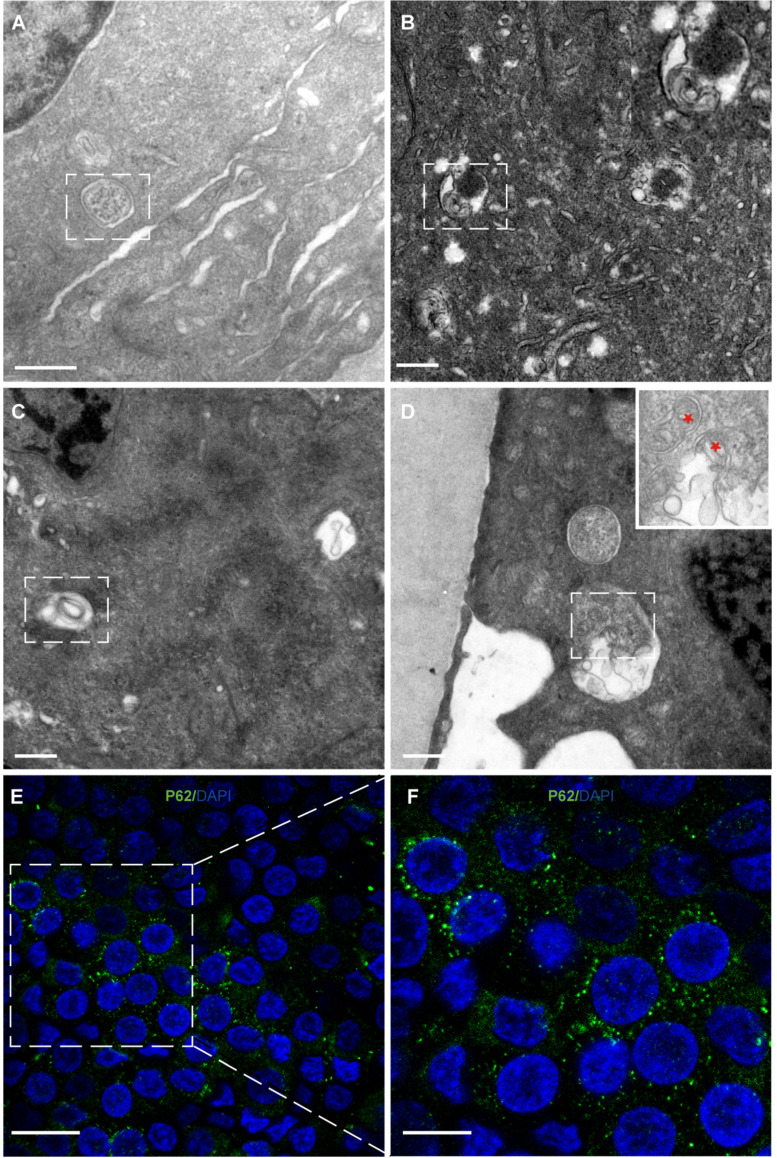
** Autophagy and mitophagy in HLECs. (A)** Autophagosome: multilamellar membranes. **(B)** Autolysosome: bilayer membrane. **(C)** Autophagic vacuole. **(D)** Mitophagy (red stars). **(E-F)** Representative images of the immunofluorescence staining of the autophagy marker P62 (green) in HLECs on the lens capsule flat-mount. Four donated human lenses: 41-year-old donor, 51-year-old donor, 41-year-old donor, and 56-year-old cataract patient **(A-D)**. Scale bars: A-D = 0.5 µm, E = 20 µm, F = 10 µm.

Autophagy and mitophagy were observed in the cytoplasm of HLECs (Figure 5A-D). Consistent with this, immunofluorescence results showed that the autophagy marker P62 was expressed in the HLECs on the lens capsule flat-mount (Figure 5E, F).

## Discussion

Knowledge of the epithelium-capsule interface and epithelium-fibre interface of the human lens helps to better understand lens physiological functions and pathological mechanisms. In the current study, by TEM and confocal microscopy, we revealed some interesting features of these structures, including capsule protrusion in some regions, initial elongating fibre cells with high staining density strongly expressing CD24, and morphological characteristics of EVs in the lens by TEM among others.

We found that the capsule protruded into the epithelium in some areas and the capsule components might be involved in potential processing. Previous studies reported the lens capsule to be a non-cellular, smooth, and transparent basement membrane secreted by HLECs and covering the surface of HLECs 10-13. However, our results showed that the human lens capsule is not smooth in some regions. Furthermore, some protruding capsules appeared as loose filament-like components with electron lucency, which might indicate phagocytosis by the epithelial cells. Such a process was found in the lens of donors aged 41, 51 and 67 years and in cataract patients aged 65 and 79 years, but not in the lens of other donors of similar age, and did not appear in the young donors or young cataract patients in the current study. It appears no obvious trends were found in the capsule protrusions in different ages of human lens and more researches are needed. These results contradict those of previous studies showing the capsule to be smooth and composed of uniformly electron-dense materials 14, 15. This specific morphological feature of the capsule in the present study might represent the progressive degradation of the protruding lens capsule, or there might be a form of communication between the capsule and epithelial cells. Many epithelia have the potential to transform into non-professional phagocytes and engulf and degrade material; for example, shedding photoreceptor outer segments are phagocytosed by retinal pigment epithelial cells to prevent debris accumulation and thereby maintain tissue homeostasis 16, 17. The question is raised whether the lens epithelial cells have the same potential of transforming into non-professional phagocytes like other epithelia. Further studies are needed to determine this.

Lens epithelial cells continuously differentiate into fibre cells, in which the organelles gradually degrade 18. Notably, the initial elongating fibre cells directly adjacent to the epithelial cells were dark staining, and appeared to correspond to the CD24-positive fibre layer on the lens vertical section. The same dense staining of this structure in TEM was also described by earlier literature and more recently by Costello et al 8, 19. A different composition and organisation of the cytoskeleton in this narrow region might contribute to this phenomenon 20. We speculated that the elongating fibre cells are just beginning to generate new cytoskeletal and cytoplasmic proteins that have not yet assembled, thus exposing more labelling sites to TEM stains. Interestingly, this structure corresponds to the CD24-positive fibre cells lens section. CD24 expression is generally high in immature precursor cells, but low or absent in terminally differentiated cells 21, 22. Previous study showed that CD24 was required for homeostatic cell renewal in the mouse, and it played an important role in the regulation of differentiated cell production through controlling the balance between cell amplification and differentiation 23. To our knowledge, CD24 expression in the human lens has not been reported before. Our study demonstrated that CD24 was selectively expressed in the young elongating fibre cells, which were adjacent to the HLECs, but not expressed in HLECs, and these results were confirmed in human lenses aged 8, 17 and 46 years (data not shown). Are these CD24+ fibre cells immature precursor fibre cells? Does it play an important role in fibre cell differentiation and could CD24, which is expressed by these fibre cells, control the formation of differentiated fibre cells? More research is required to answer these questions.

The current study provided clear evidence that numerous EVs appear between adjacent epithelial cells and between the capsule and epithelium in the human lens, and demonstrated their morphological features by TEM. EVs are being identified in an increasing number of cell types and it was not surprising to find them in the human lens epithelium. Although EVs have been previously isolated from the aqueous humour of cataract patients and HLEC cell lines 24-26, to our knowledge, such an extensive presence of small EVs has not previously been reported for the human lens epithelium. EVs play an important role in the communication between cells in both, physiological and pathological processes 27-29. EVs are generally considered to contain various molecules, including messenger molecules, enzymes, RNAs, and DNA fragments 30. The morphology of EVs observed in this study appeared to be diversity, which consists of four types of the EVs: (1) outward budding: small separate EVs were generated in the cytoplasm and secreted into the outward budded plasma membrane, forming the EV complex (Figure 4A, E); (2) shedding: the EV complex was shed from the epithelial cells (Figure 4B, F); (3) release: the vesicles were released into the intercellular space (Figure 4C, G); (4) contact: released EVs became bound to the target epithelial cell or capsule (Figure 4D, H). Due to the limitation of 2D images in this study, however, we could not exclude a possibility that these types of EVs might be from different views of the same type of cluster. This question could be revealed by three-dimensional EM work in the future. In addition, EVs in the cataract samples appeared to be more irregular and disorganised, and the four typical morphologies as mentioned above were rarely observed. Further study is needed to evaluate the significance of these findings.

The diameter of EVs found in our study ranged from 26.8 to 307.3 nm, and the majority (approximately 83.8%) were 30-150 nm, indicating that these EVs might have been exosomes. Furthermore, the immunofluorescence results showed that two exosome biomarkers (CD63 and CD81) were expressed in the HLECs on the lens capsule flat-mount. Taken together, we suggest that there might be exosomes in the extracellular space of the HLECs. However, further evidence, TEM on isolated tissue and immunogold TEM with specific markers for exosomes, is needed.

Literatures showed that autophagy and mitophagy are required for ocular lens differentiation and transparency 31. And in current study, autophagy and mitophagy were observed in the HLECs, which is consistent with previous study.

The limitation of this study is that the selected samples were mainly from donated eyes, which were still somewhat different from normal eyes. Second, although we attempted to use lenses without systemic diseases, it was still not completely certain whether the donors were under certain stress or an unknown pathological state premortally.

In summary, the current study provides some interesting ultrastructural information of the interface of the epithelium with the lens capsule and fibre cell, which might help to achieve a deeper understanding of the physiological function and pathological mechanism of the human lens.

## Figures and Tables

**Table 1:** Lens information of the donors and cataract patients

Donors	1	2	3	4	5	6	7	8	9	10	11	12
Age	16 month	8y	17y	36y	41y	46y	51y	51y	53y	55y	64y	67y
Gender	M	M	M	M	M	M	M	F	M	M	M	M
EVs	yes	yes	yes	yes	yes	yes	yes	yes	yes	yes	yes	yes
Capsule Protrusion	no	no	no	no	yes	no	yes	no	no	no	no	yes
Cataract patients	1	2	3	4	5	6	7	8	9	10	11	12
Age	2y	5y	5y	19y	23y	56y	65y	67y	69y	70y	72y	79y
Gender	M	F	M	M	M	F	F	F	F	M	F	M
Clinical diagnosis	Congenital cataract	Congenital cataract	Congenital cataract	Complicated cataract	Complicated cataract	ARC	Complicated cataract	ARC	ARC	ARC	Complicated cataract	ARC
EVs	yes	yes	yes	no	yes	yes	yes	yes	yes	no	yes	no
Capsule Protrusion	no	no	no	no	no	no	yes	no	no	no	no	yes

ARC, Age-related cataract; EVs, Extracellular vesicles

## References

[1] Morishita H, Mizushima N (2016). Autophagy in the lens. Exp Eye Res.

[2] Flaxman SR, Bourne RRA, Resnikoff S, Ackland P, Braithwaite T, Cicinelli MV (2017). Global causes of blindness and distance vision impairment 1990-2020: a systematic review and meta-analysis. Lancet Glob Health.

[3] Cvekl A, McGreal R, Liu W (2015). Lens Development and Crystallin Gene Expression. Prog Mol Biol Transl Sci.

[4] Zampighi GA, Eskandari S, Kreman M (2000). Epithelial organization of the mammalian lens. Exp Eye Res.

[5] Francois J, Victoria-Troncoso V (1978). Histology of the epithelium of the normal and cataractous lens. Ophthalmologica.

[6] Broglio TM, Worgul BV (1982). The lens epithelium and radiation cataract. IV. Ultrastructural studies of interphase death in the meridional rows. Virchows Arch B Cell Pathol Incl Mol Pathol.

[7] Farnsworth PN, Burke-Gadomski P, Kulyk T, Mauriello JA, Cinotti AA (1976). Surface ultrastructure of the epithelial cells of the mature human lens. Exp Eye Res.

[8] Costello MJ, Mohamed A, Gilliland KO, Fowler WC, Johnsen S (2013). Ultrastructural analysis of the human lens fiber cell remodeling zone and the initiation of cellular compaction. Exp Eye Res.

[9] Taylor VL, al-Ghoul KJ, Lane CW, Davis VA, Kuszak JR, Costello MJ (1996). Morphology of the normal human lens. Invest Ophthalmol Vis Sci.

[10] Ronci M, Sharma S, Chataway T, Burdon KP, Martin S, Craig JE (2011). MALDI-MS-imaging of whole human lens capsule. J Proteome Res.

[11] Huang D, Xu C, Guo R, Ji J, Liu W (2020). Anterior lens capsule: biomechanical properties and biomedical engineering perspectives. Acta Ophthalmol.

[12] Tholozan FM, Gribbon C, Li Z, Goldberg MW, Prescott AR, McKie N (2007). FGF-2 release from the lens capsule by MMP-2 maintains lens epithelial cell viability. Mol Biol Cell.

[13] Blakely EA, Bjornstad KA, Chang PY, McNamara MP, Chang E, Aragon G (2000). Growth and differentiation of human lens epithelial cells *in vitro* on matrix. Invest Ophthalmol Vis Sci.

[14] Straatsma BR, Lightfoot DO, Barke RM, Horwitz J (1991). Lens capsule and epithelium in age-related cataract. Am J Ophthalmol.

[15] Marshall GE, Konstas AG, Bechrakis NE, Lee WR (1992). An immunoelectron microscope study of the aged human lens capsule. Exp Eye Res.

[16] Strauss O (2005). The retinal pigment epithelium in visual function. Physiol Rev.

[17] Chen X, Guo F, LeBlanc ME, Ding Y, Zhang C, Shakya A (2016). Mesd extrinsically promotes phagocytosis by retinal pigment epithelial cells. Cell Biol Toxicol.

[18] Bassnett S (2002). Lens organelle degradation. Exp Eye Res.

[19] Costello MJ, Brennan LA, Basu S, Chauss D, Mohamed A, Gilliland KO (2013). Autophagy and mitophagy participate in ocular lens organelle degradation. Exp Eye Res.

[20] Wenke JL, McDonald WH, Schey KL (2016). Spatially Directed Proteomics of the Human Lens Outer Cortex Reveals an Intermediate Filament Switch Associated with the Remodeling Zone. Invest Ophthalmol Vis Sci.

[21] Wenger RH, Rochelle JM, Seldin MF, Kohler G, Nielsen PJ (1993). The heat stable antigen (mouse CD24) gene is differentially regulated but has a housekeeping promoter. J Biol Chem.

[22] Fang X, Zheng P, Tang J, Liu Y (2010). CD24: from A to Z. Cellular & molecular immunology.

[23] Nieoullon V, Belvindrah R, Rougon G, Chazal G (2007). Mouse CD24 is required for homeostatic cell renewal. Cell Tissue Res.

[24] Gao C, Fan F, Liu X, Yang J, Zhou X, Mei H (2020). Exosomal miRNA Analysis of Aqueous Humour of Diabetes and Cataract Patients. Curr Eye Res.

[25] Wang R, Li J, Zhang X, Zhang X, Zhang X, Zhu Y (2021). Extracellular vesicles promote epithelial-to-mesenchymal transition of lens epithelial cells under oxidative stress. Exp Cell Res.

[26] Dismuke WM, Challa P, Navarro I, Stamer WD, Liu Y (2015). Human aqueous humor exosomes. Exp Eye Res.

[27] Voigt AP, Binkley E, Flamme-Wiese MJ, Zeng S, DeLuca AP, Scheetz TE (2020). Single-Cell RNA Sequencing in Human Retinal Degeneration Reveals Distinct Glial Cell Populations. Cells.

[28] Liu M, Wang D, Gu S, Tian B, Liang J, Suo Q (2021). Micro/nano materials regulate cell morphology and intercellular communication by extracellular vesicles. Acta Biomater.

[29] Zieske JD, Hutcheon AEK, Guo X (2020). Extracellular Vesicles and Cell-Cell Communication in the Cornea. Anat Rec (Hoboken).

[30] Somiya M (2020). Where does the cargo go?: Solutions to provide experimental support for the "extracellular vesicle cargo transfer hypothesis". J Cell Commun Signal.

[31] Brennan L, Costello MJ, Hejtmancik JF, Menko AS, Riazuddin SA, Shiels A (2023). Autophagy Requirements for Eye Lens Differentiation and Transparency. Cells.

